# Self-reported adherence supports patient preference for the single tablet regimen (STR) in the current cART era

**DOI:** 10.7448/IAS.15.6.18104

**Published:** 2012-11-11

**Authors:** G Sterrantino, L Santoro, M Trotta, A Antinori, D Bartolozzi, M Zaccarelli

**Affiliations:** 1 Azenda Ospedaliera Universitaria Careggi, Malattie Infettive, Florence, Italy; 2 Istituto Nazionale per le Malattie Infettive “Lazzaro Spallanzani”, III Divisione di Malattie Infettive, Rome, Italy; 3 Istituto Nazionale per le Malattie Infettive “Lazzaro Spallanzani”, Unita' Operativa Immunodeficienze Virali, Rome, Italy

## Abstract

**Objective:**

To analyze self-reported adherence to antiretroviral regimens containing ritonavir-boosted protease inhibitors, non-nucleoside reverse transcriptase inhibitors (NNRTI), raltegravir, and maraviroc.

**Methods:**

Overall, 372 consecutive subjects attending a reference center for HIV treatment in Florence, Italy, were enrolled in the study, from December 2010 to January 2012 (mean age 48 years). A self-report questionnaire was filled in. Patients were defined as “non-adherent” if reporting one of the following criteria:<90% of pills taken in the last month, ≥1 missed dose in the last week, spontaneous treatment interruptions reported, or refill problems in the last 3 months. Gender, age, CD4, HIV-RNA, years of therapy, and type of antiretroviral regimen were analyzed with respect to adherence.

**Results:**

At the time of the questionnaire, 89.8% of patients had <50 copies/mL HIV-RNA and 14.2% were on their first combined antiretroviral therapy. 57% of patients were prescribed a regimen containing ritonavir boosted protease inhibitors (boosted PI), 41.7% NNRTI, 17.2% raltegravir, and 4.8% maraviroc; 49.5% of the subjects were on bis-in-die regimens, while 50.5% were on once-daily regimens, with 23.1% of these on the single tablet regimen (STR): tenofovir/emtricitabine/efavirenz. The non-adherence proportion was lower in NNRTI than in boosted-PI treatments (19.4% vs 30.2%), and even lower in STR patients (17.4%). In multivariable logistic regression, patients with the NNRTI regimen (OR: 0.56, 95% CI: 0.34–0.94) and the STR (OR: 0.45, 95% CI: 0.22–0.92) reported lower non-adherence. Efavirenz regimens were also associated with lower non-adherence (OR: 0.42, 95% CI: 0.21–0.83), while atazanavir/ritonavir regimens were associated with higher non-adherence. No other relation to specific antiretroviral drugs was found. A higher CD4 count, lower HIV-RNA, and older age were also found to be associated with lower non-adherence, while a longer time on combined antiretroviral therapy was related to higher non-adherence.

**Conclusion:**

In conclusion, older age, higher CD4 cell counts, lower HIV-RNA viral loads, and the use of STR are all related to lower non-adherence. In particular, the use of STR maintains an advantage in improving adherence with respect to other cARTs, even with the availability of new, well-tolerated antiretroviral drugs and drug classes in recent years.

